# Residual Safety Margin-Based Risk Stratification for Hospital-Wide POCT Glucose Meters Anchored to ISO 15197: Moving Beyond Pass-Fail

**DOI:** 10.3390/diagnostics15243220

**Published:** 2025-12-16

**Authors:** Hao Bi, Yuting Chen, Yihan Wu, Zuliang Shi, Jianbo Xia, Qiuyue Yan

**Affiliations:** Department of Laboratory Medicine, Maternal and Child Health Hospital of Hubei Province, Tongji Medical College, Huazhong University of Science and Technology, Wuhan 430070, China; bihao@hbfy.com (H.B.);

**Keywords:** POCT, glucose meters, quality management, ISO 15197:2013, residual safety margin, risk stratification, TEa, hospital governance

## Abstract

**Background**: In this hospital-wide evaluation of point-of-care testing (POCT) glucose meters, we introduced a residual safety margin (*r*) anchored to ISO 15197:2013 thresholds to quantify tolerance, move beyond binary pass/fail assessments, and enable risk stratification. **Methods**: Thirty-five departmental glucose meters were compared with a central laboratory reference at five predefined glucose concentrations. Compliance was assessed using ISO 15197:2013 point-wise limits, Bland–Altman analysis was used to estimate bias and limits of agreement, and the mean absolute relative difference (MARD) and root mean square error (RMSE) were calculated to summarize overall error. At each concentration, *r* was calculated for every department, ranked, and classified into low, medium, or high risk using allowable error thresholds based on biological variation, specifically total allowable error (TEa), mapped to the ISO limits. **Results**: All departments met ISO criteria (100% compliance; 95% CI: 97.9–100%). Mean bias was −1.43 mg/dL, with limits of agreement from −15.6 to 12.8 mg/dL; MARD was 3.8% (95% CI: 3.4–4.3%), and RMSE was 7.4 mg/dL (95% CI: 6.6–8.2 mg/dL). Despite universal compliance, r-based analysis revealed concentration-related heterogeneity and highlighted borderline-performing departments that were overlooked by conventional metrics. **Conclusions**: By anchoring residual safety margins to ISO thresholds, the r framework shifts POCT glucose assessment from a binary pass/fail decision to a risk-stratified ranking approach, exposing latent performance variation and supporting targeted quality improvement at the hospital level.

## 1. Introduction

Point-of-care testing (POCT) for blood glucose is widely adopted for clinical glucose monitoring and control, from emergencies to routine ward care, due to its minimal blood volume requirement and rapid turnaround time [1]. However, POCT glucose systems are susceptible to analytic and pre-analytic errors, so robust quality assurance is essential [2]. In hospitalized patients, POCT results often guide insulin dosing and other critical decisions, and even small deviations may have important clinical consequences [3]. In hospital wide comparison studies, POCT performance is commonly assessed by analyzing five whole blood samples at predefined glucose concentrations, comparing the results with a laboratory reference, and evaluating compliance with ISO 15197:2013 criteria, which defines allowable limits of ±15 mg/dL for glucose values below 100 mg/dL and ±15% for values at or above 100 mg/dL [4].

However, this compliance framework typically yields only a binary outcome—pass or fail—that confirms whether minimum standards are met but conceals important nuances in performance relative to the thresholds. Some departments operate well within the limits, while others approach the boundary, where minor fluctuations may result in non-compliance [5]. In such scenarios, binary pass/fail evaluations provide insufficient information for hospital-level risk stratification or quality governance.

Traditional agreement methods, including correlation analysis, Bland–Altman (BA) plots, and Passing–Bablok or Deming regression, support method comparison but offer limited utility for hospital-wide, cross-departmental risk stratification. Correlation summarizes global linear association but may overstate agreement in the presence of systematic bias. BA visualizes bias and variability, yet does not assess compliance with ISO-defined acceptability limits [6,7]. Aggregate error metrics, such as the mean absolute relative difference (MARD) and root mean square error (RMSE), are informative but cannot replace point-specific ISO evaluations [8]. While Passing–Bablok regression can reveal department-specific systematic bias (intercept/slope), it does not directly assess compliance and therefore lacks sufficiency for risk-based quality management [9].

To address the above limitations, the ISO anchored residual safety margin *r* was introduced as a conceptual framework to quantify the remaining safety buffer between observed values and allowable error. Rather than yielding a binary classification of “pass” or “fail”, *r* enables concentration-specific, department-level risk stratification and near-threshold detection that can be acted upon within a quality-governance loop and is congruent with the risk-based emphasis in ISO 15189 revisions [10,11,12]. In this hospital-wide POCT evaluation, the *r* framework was applied to five predefined glucose concentrations, allowing stratification of residual risk across departments at each level. This method enabled clearer prioritization of quality improvement efforts, supported data-driven decisions, and facilitated continuous monitoring and periodic reassessment.

## 2. Materials and Methods

### 2.1. Design and Setting

This study was conducted at a large tertiary Maternal and Child Health hospital and included 35 clinical departments, each with one POCT glucose meter. Devices were from three different brands, and for each brand, one lot number was used throughout the hospital to minimize inter-lot variability. EDTA-anticoagulated whole blood was used, with five predefined reference glucose levels: 41, 82, 123, 227, and 291 mg/dL. Each device performed triplicate measurements at each level, and the mean was compared with the central laboratory reference (Beckman Coulter AU5800; glucose oxidase) [4]. Detailed information is summarized in Table 1 and Table S1.

### 2.2. Hospital-Wide POCT Comparison Quality Assurance

All POCT in the hospital-wide comparison followed a unified institutional quality assurance (QA) system. Each device had completed method verification; operators were certified after standardized training and competency checks. On the study day, test strips and quality-control (QC) materials were in date and handled per the instructions for use (IFU) under specified environmental conditions. Recent external quality assessment (EQA) met program criteria, and two levels of internal quality control (IQC) were within range. Sample-stability requirements and exclusion of invalid specimens followed ISO 15197:2013.

### 2.3. Endpoints and Calculations

Primary analytical endpoints included: ISO 15197:2013 per-point acceptability [4], BA mean bias, and 95% limits of agreement (LOA), MAR, and RMSE [7,8]. Statistical methods follow standard references.

*r* was calculated as a new metric: *r* = max {0, 1–use}, where use = |bias|/ISO limit. Here, bias is defined for each department at each reference level as the difference between the mean of three POCT replicates and the paired reference value: Bias (mg/dL) = mean (POCT_1_, POCT_2_, POCT_3_) − Reference (mg/dL). ISO 15197:2013 acceptance limits are 15 mg/dL when the reference value is <100 mg/dL and 0.15 × the reference value when it is ≥100 mg/dL. Because r is calculated separately at each reference level using these absolute (<100 mg/dL) and relative (≥100 mg/dL) ISO limits, it does not rely on an assumption of constant variance across the measurement range.

At five reference levels, department-level *r* values were calculated and ranked. Two threshold lines were defined under the EFLM/BV allowable-error framework: total allowable error (TEa) (≤54 mg/dL: 3.78 mg/dL; >54 mg/dL: 7% of the reference) and half-TEa (≤54 mg/dL: 1.89 mg/dL; >54 mg/dL: 3.5% of the reference), consistent with management approaches based on allowable error and sigma-metrics [13,14]. Using these cutoffs, departments at each level were classified as high risk (*r* < r*(TEa)), medium risk (r*(TEa) ≤ *r* < r*(1/2TEa)), or low risk (*r* ≥ (r*1/2)TEa). Here, r*(TEa) = 1 − TEa limit/ISO limit and r*(1/2TEa) = 1 − half-TEa limit/ISO limit.

### 2.4. Statistical Analysis

All statistical analyses and visualizations were performed using R (version 4.5.1). ISO 15197:2013 per-point compliance was estimated with two-sided Wilson 95% confidence intervals. Confidence intervals for BA limits of agreement, MARD, and RMSE were calculated using a two-sided percentile bootstrap approach (2000 resamples); when observations were nested within departments, a clustered bootstrap by department was used [15].

## 3. Results

### 3.1. Overall Agreement and Accuracy Assessment

**Figure 1:**
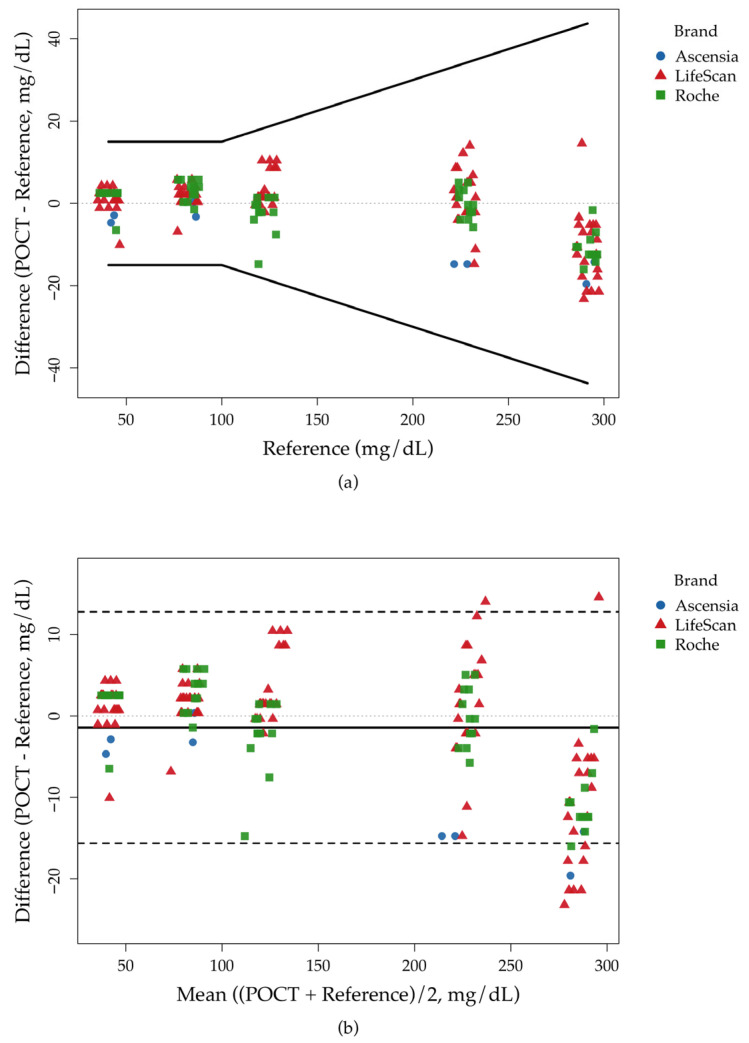
Agreement of POCT glucose with the laboratory reference across 175 paired measurements. (**a**) Per-point acceptability relative to ISO 15197:2013 allowable-error limits (reference < 100 mg/dL: ±15 mg/dL; ≥100 mg/dL: ±15%). Differences are plotted against the reference value; the dashed lines represent perfect agreement (y = x), and solid lines indicate the ISO 15197:2013 allowable-error limits. (**b**) BA plot showing the mean bias (−1.43 mg/dL) and 95% limits of agreement (LoA) (−15.6 to 12.8 mg/dL). The solid line represents the mean bias, and the dashed lines indicate the 95% LoA. Abbreviations: POCT, point-of-care testing.

A total of 175 paired measurements were analyzed across all departments. The per-point compliance with ISO 15197:2013 was 100.0% (95% CI: 97.9–100.0%). BA analysis showed a mean bias of −1.43 mg/dL, with 95% LoA from −15.6 to 12.8 mg/dL. Overall, MARD was 3.8% (95% CI: 3.4–4.3%), and the RMSE was 7.4 mg/dL (95% CI: 6.6–8.2 mg/dL) (Table 2 and Figure 1a). Brand-level estimates are provided in Supplementary Table S2. As shown in Figure 1b, differences clustered around the BA mean line (−1.43 mg/dL). Dispersion increased at higher concentrations (227 and 291 mg/dL), and at 291 mg/dL, biases shifted more negative with a few points near or slightly beyond the lower LoA. These patterns indicate greater error at higher glucose levels, although all observations remained within ISO per-point acceptability limits.

### 3.2. Performance and Agreement Across Glucose Concentration Levels

**Figure 2:**
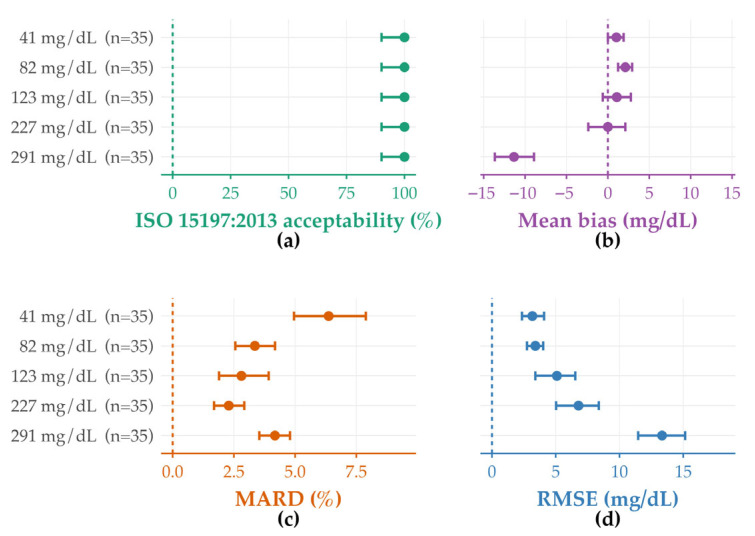
Concentration-stratified analytical performance at five predefined reference glucose levels. (**a**) ISO 15197:2013 per-point acceptability (Wilson 95% CIs). (**b**) Bland–Altman mean bias (POCT − reference) by level with percentile-bootstrap 95% CIs (B = 2000). (**c**) MARD (%) by level with percentile-bootstrap 95% CIs (B = 2000). (**d**) RMSE (mg/dL) by level with percentile-bootstrap 95% CIs (B = 2000).

Across the five reference glucose levels (41, 82, 123, 227, and 291 mg/dL), BA analysis by level (see Figure 2 and Supplementary Table S3) showed a slight positive bias at 41 mg/dL, similarly mild positive bias at 82 and 123 mg/dL, near-zero mean bias at 227 mg/dL (95% CI crossing zero), and a more pronounced negative bias at 291 mg/dL (Figure 2b). Per-point ISO 15197:2013 acceptability was 100% at every level (Figure 2a). MARD followed the expected U-shape: lowest at 82–123 mg/dL (~2–3%) and higher at the extremes (~6–8% at 41 mg/dL; ~3–5% at 291 mg/dL; Figure 2c). RMSE (absolute error, mg/dL) increased with concentration (Figure 2d). In sum, all levels met ISO criteria; absolute and relative errors were smallest in the low-to-mid range, while absolute error rose at higher concentrations, but relative error remained acceptable.

### 3.3. Department-Level Risk Stratification by ISO-Anchored r with TEa-Referenced Bands

**Figure 3:**
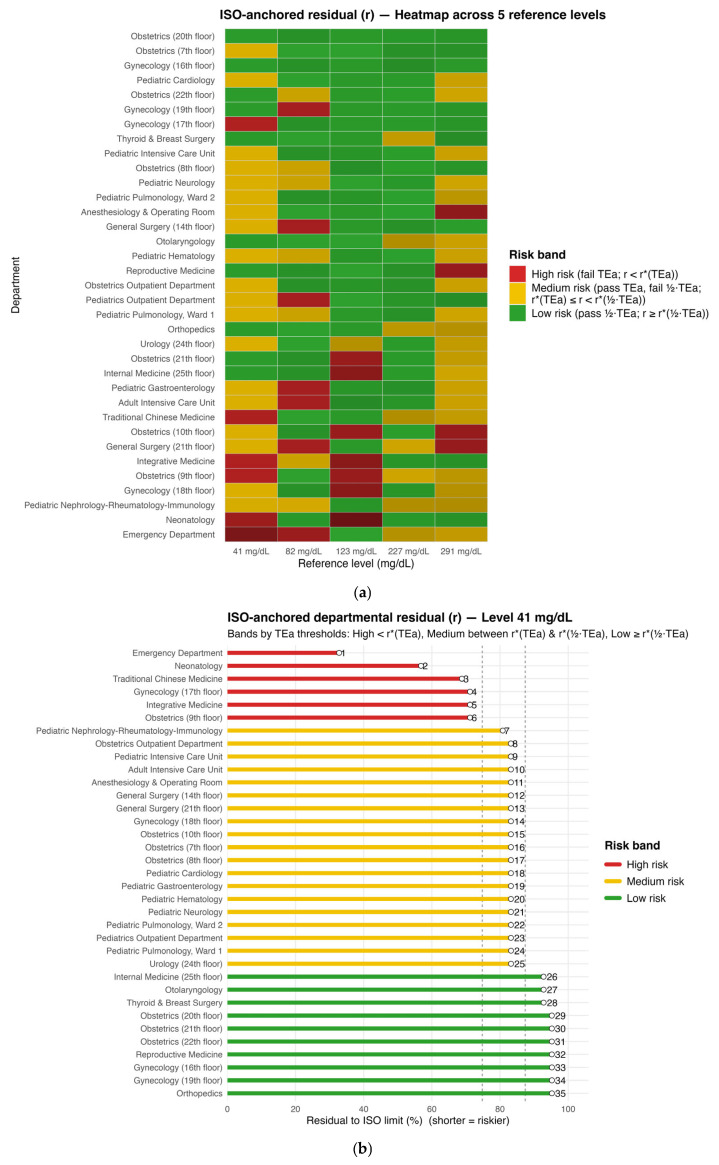

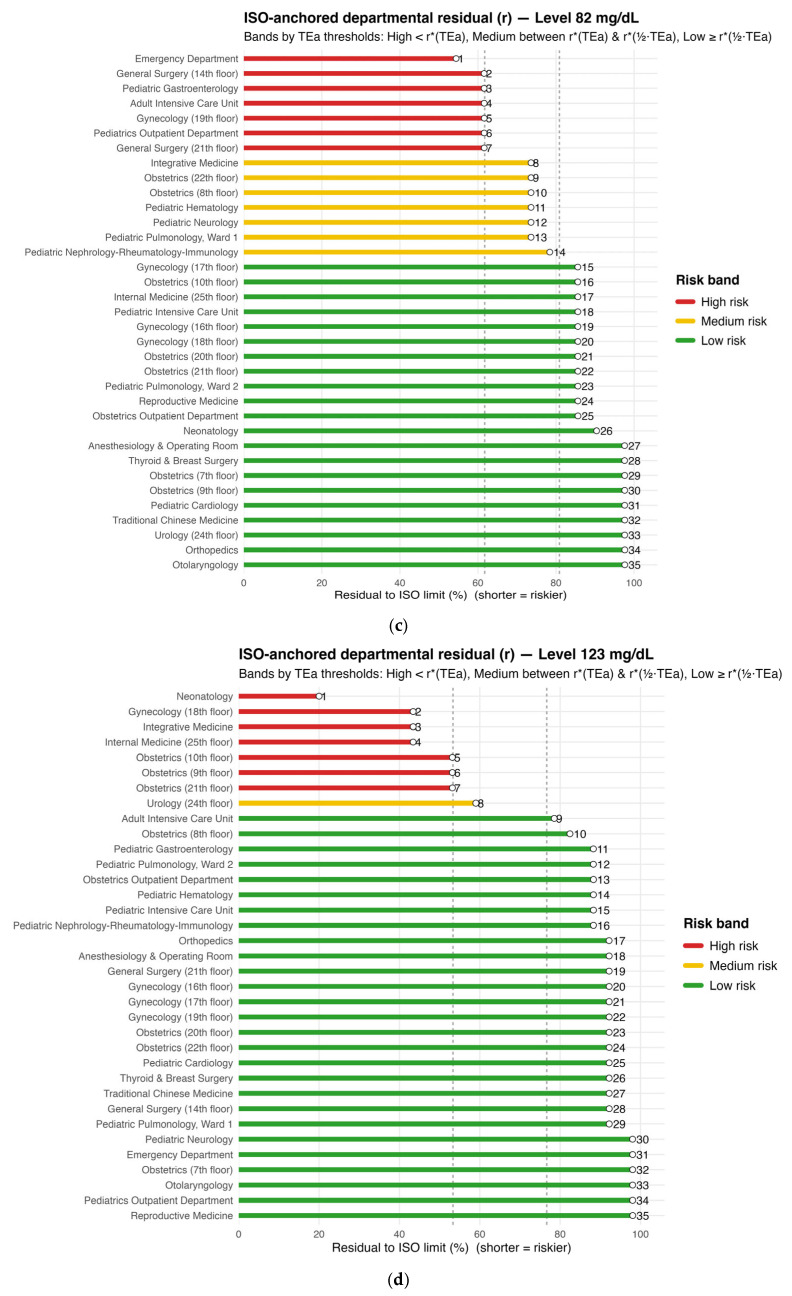

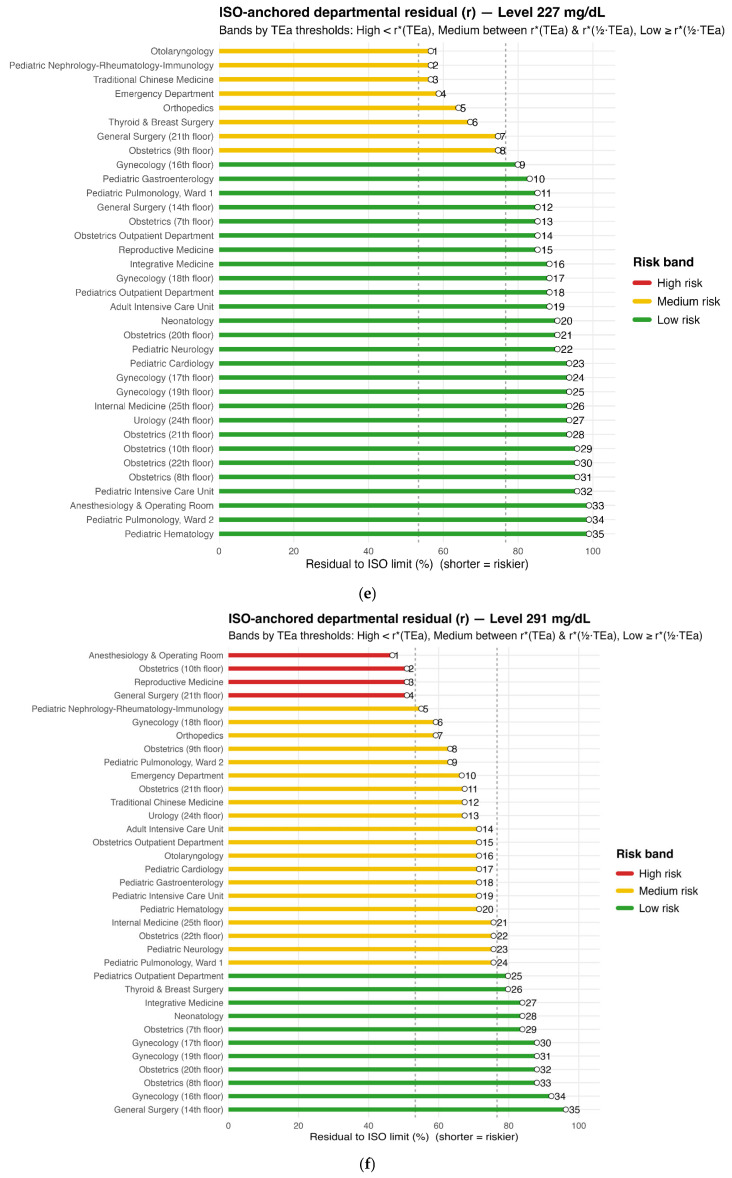
Department-level risk stratification by ISO-anchored residual *r*. (**a**) Heatmap of department-level bands across five reference glucose levels (41, 82, 123, 227, and 291 mg/dL). Colors denote risk bands: high (red, *r* < r*(TEa)), medium (yellow, r*(TEa) ≤ *r* < r*(1/2TEa)), and low (green, *r* ≥ r*(1/2TEa)); (**b**–**f**) Departmental rankings at each level: (**b**) 41 mg/dL; (**c**) 82 mg/dL; (**d**) 123 mg/dL; (**e**) 227 mg/dL; and (**f**) 291 mg/dL. Bars show 100 × *r*; vertical dashed lines indicate r*(TEa) and r*(1/2TEa).

Across the five reference levels, concentration dependence and inter-department variability in *r* were evident. Pediatric Neurology was classified as low at most levels, whereas the Emergency Department more often fell into medium/high (Figure 3a). At the low reference levels (41 and 82 mg/dL), the ISO-anchored *r* heat map (Figure 3a) flagged several departments as medium or high risk with only small residual safety margins near the TEa-based thresholds, even though the pooled Bland–Altman plot (Figure 1b) suggested overall acceptable agreement. Departmental rankings at each level are shown in Figure 3b–f. Department-level *r* values and per-level ranks are provided in Supplementary Table S4a–e.

## 4. Discussion

In this single-center evaluation of 35 POCT glucose meters across five predefined levels, all devices met the per-point ISO 15197:2013 acceptance criteria under standardized conditions, indicating acceptable system accuracy. With concentration stratification, RMSE increased with glucose level, whereas MARD was lowest in the mid-range and slightly higher at both ends [8]. At higher concentrations, a shift toward more negative bias and wider dispersion was observed [5,16]. Taken together, these findings suggest that, when pre-analytical steps are controlled and staff are trained, POCT glucose testing can approach central-laboratory accuracy across departments [17,18].

Traditional tools such as Bland–Altman plots and Passing–Bablok regression remain essential for method comparison but are limited for cross-department governance: they use pooled data, do not map directly to ISO point-wise limits, and give little weight to near-threshold risk [4,6,19]. To address this, we introduced an ISO-anchored residual safety margin *r*, calculated separately at five reference levels. This level-specific *r* is directly tied to ISO 15197 limits, separates low/high from mid-range performance, is more sensitive to near-limit risk, allows cross-department ranking at each level, and, when combined with TEa and 1/2TEa, maps results into actionable red/yellow/green risk bands, while the continuous r ∈ [0, 1] scale supports threshold setting and trend tracking [13,20]. At low reference levels (41 and 82 mg/dL), for example, the pooled Bland–Altman plot (Figure 1b) still suggests good overall agreement, whereas the ISO-anchored r heat map (Figure 3a) flags several departments as medium or high risk with only narrow residual safety margins to the TEa-based thresholds, revealing level-specific critical risk that is not apparent from the pooled plot alone. At higher concentrations, dispersion increases and bias shifts more negative, yet because r is defined separately at each reference level and anchored to the absolute (<100 mg/dL) and relative (≥100 mg/dL) ISO limits, its clinical interpretation remains straightforward across the range, and the per-level heatmaps and rankings further facilitate communication and implementation in hospital quality-management practice.

Applied to the hospital-wide dataset, *r*-based stratification revealed heterogeneity in residual safety margins despite overall ISO compliance, consistent with prior multi-system evaluations showing substantial between-device variability even when ISO criteria are used as the yardstick [5]. From the heatmap and the risk-stratification plots at the five reference levels, the mid-range was predominantly low, whereas medium/high occurred more often at the extremes (41 and 291 mg/dL), in line with reports that absolute error tends to rise with concentration, while MARD is lowest around the mid-range [8]. Mechanistically, low-level anomalies are compatible with zero-point offset/limited sensitivity/background drift and matrix effects, all known contributors to bias at low glucose [21]. In contrast, high-level deviations can reflect amplified proportional bias and upper-range nonlinearity/saturation, and performance can deteriorate under temperature/humidity stress, which increases dispersion—practical concerns for ward-based POCT [22]. Occasional mid-range departures are consistent with slope–intercept mismatch on method comparison, which is routinely characterized via Passing–Bablok/Deming frameworks [23].

Accordingly, remediation should be level-specific and device-focused: at low levels, prioritize zero-point checks/adjustment and denser checks near the lower end, supported by strengthened IQC; at mid-levels, perform traceability checks and recalibrate slope and intercept (with confidence intervals verified by method-comparison regression); and at high levels, consider segmented or nonlinear calibration, add denser upper-range checks, and verify environmental robustness (temperature/power stability), escalating to vendor module evaluation when needed. These steps are consistent with contemporary guidance on quality indicators/sigma-metrics for continuous improvement in laboratory medicine [12,13], and with recent POCT-specific frameworks that use process mapping and risk-based selection of quality indicators for glucose point-of-care testing [24,25], as well as with evidence that comparison-method choice influences apparent accuracy, hence the need for traceability verification [12].

For hospital-level governance, POCT comparisons should go beyond a binary pass/fail and proceed in four layers: first, apply ISO single-point acceptability as the compliance gate; second, conduct performance evaluation using BA (mean bias and limits of agreement), PB regression (slope/intercept with confidence intervals), MARD, and RMSE, followed by concentration-stratified review that assesses absolute-error impact at low levels and dispersion plus negative-bias risk at high levels to locate the problematic ranges; third, implement *r*-based departmental banding, using green/yellow/red to quantify distance to the ISO boundary and enable within-level comparability and risk stratification; finally, for high-risk devices, define concentration-specific corrective actions and verify their effectiveness to complete a closed improvement loop. Within this framework, pooled BA, PB regression, MARD, and RMSE describe overall system performance, while r provides an ISO-anchored, department-level risk banding for governance.

This study has limitations: single center, conducted under controlled workflows, and used a single lot, which may underestimate real-world variability. Follow-up work will apply this ISO-anchored framework across departments, lots, and time within department-specific representative concentration ranges, re-evaluate the discriminative performance of *r*, and longitudinally assess its impact on IQC and external quality assessment/proficiency testing (EQA/PT) performance. In this initial implementation, brand-level results (including Ascensia, n = 10) are reported as descriptive only; future applications that specifically focus on between-brand comparisons will predefine simple minimum sample-size and confidence-interval-width criteria before brand rankings are used to inform governance decisions.

In summary, an ISO-anchored residual safety margin *r*, used within a TEa-referenced framework, enables an assessment that goes beyond pass/fail. Even when ISO criteria are met, safety margins can be stratified and ranked to expose near-threshold risks and heterogeneity (by concentration, department, and device), turning “compliant” into a comparable risk gradient with clear priorities. This approach uses appropriate statistical characterization (e.g., *r*-based risk banding) to identify potential issues in advance and to guide precision quality control and continuous improvement.

## Figures and Tables

**Table 1:** Characteristics of POCT blood glucose monitoring systems and the laboratory reference analyzer.

Brand (Manufacturer, Country)	Departments (n)	Assay Chemistry (Strip/Enzyme)	Measurement Range (mg/dL)	Hematocrit Range (%)	Operating Temperature (°C)	Operating Humidity (%)	Strip/ Reagent Lot	Method Type
LifeScan OneTouch Verio (LifeScan, Malvern, PA, USA)	21	FAD-dependent glucose dehydrogenase (GDH-FAD)	20–600	20–60	15–40	10–90	5869580	POCT
Roche Accu-Chek Inform II (Roche Diagnostics, Mannheim, Germany)	12	Mutant quinoprotein glucose Dehydrogenase (Mut. Q-GDH; maltose-independent)	11–600	10–65	8–44	10–90	671596	POCT
Ascensia CONTOUR TS (Ascensia Diabetes Care, Basel, Switzerland)	2	FAD-dependent glucose dehydrogenase (GDH-FAD)	20–600	15–65	5–45	10–93	DP4HM3F31E	POCT
Beckman Coulter AU5800 (Beckman Coulter Diagnostics, Brea, CA, USA)	1	Glucose oxidase (GOD)	10–600	N/A	18–32	30–80	25030502	Reference analyzer

Abbreviations: FAD = flavin adenine dinucleotide; GDH-FAD = FAD-dependent glucose dehydrogenase; Mut. Q-GDH = mutant quinoprotein glucose dehydrogenase; GOD = glucose oxidase; POCT = point-of-care testing; N/A = not applicable.

**Table 2:** Overall accuracy and agreement with the laboratory reference.

Pairs (n)	ISO 15197, % [95% CI]	BA Mean Bias, mg/dL (95% LoA)	MARD, % [95% CI]	RMSE, mg/dL [95% CI]
175	100.0 [97.9, 100.0]	−1.43 (−15.6, 12.8)	3.8 [3.4, 4.3]	7.4 [6.6, 8.2]

## Data Availability

De-identified data supporting the findings of this study are available from the corresponding author upon reasonable request.

## Supplementary Materials

The following supporting information can be downloaded at: https://www.mdpi.com/article/10.3390/diagnostics15243220/s1: Table S1: Distribution of participating departments by POCT glucose meter brand; Table S2: Brand-level accuracy and agreement with the laboratory reference (mg/dL); Table S3: Per-level analytical performance of POCT glucose versus the laboratory reference; Table S4a–e: Department-level r-based performance rankings at five reference levels.

## Abbreviations

The following abbreviations are used in this manuscript:

BA	Bland–Altman
BV	Biological variation
CI	Confidence interval
CV	Coefficient of variation
EFLM	European Federation of Clinical Chemistry and Laboratory Medicine
EQA	External quality assessment
FAD	Flavin adenine dinucleotide
GDH-FAD	FAD-dependent glucose dehydrogenase
GOD	Glucose oxidase
IFU	Instructions for use
IQC	Internal quality control
ISO	International Organization for Standardization
LoA	Limits of agreement
MARD	Mean absolute relative difference
Mut. Q-GDH	Mutant quinoprotein glucose dehydrogenase
POCT	Point-of-care testing
QC	Quality control
QA	Quality assurance
RMSE	Root mean square error
SD	Standard deviation
TEa	Total allowable error
