# Supplementary material for: Residual Safety Margin-Based Risk Stratification for Hospital-Wide POCT Glucose Meters Anchored to ISO 15197: Moving Beyond Pass-Fail
Source: Diagnostics (Basel). 2025 Dec 16;15(24):3220. doi: 10.3390/diagnostics15243220 (PMC12731778; doi:10.3390/diagnostics15243220)
Supplement: Supplementary file 1 [file diagnostics-15-03220-s001.zip › diagnostics-3985486-supplementary.pdf]

Supplementary Materials

Table S1. Distribution of participating departments by POCT glucose meter brand

System	Brand	Departments (abbrev., n = per system)
A	LifeScan OneTouch Verio (LifeScan, USA)	(n=21), RM; ORTH; OB-22F; OB-21F; OB-20F; OB-10F; OB-9F; OB-8F; OB-7F; OB-OPD; IM-25F; IMed; GYN-19F; GYN-18F; GYN-17F; GYN-16F; TBS; ENT; ED; GS-21F; ANES/OR
B	Roche Accu-Chek Inform II (Roche Diagnostics, Germany)	(n=12), P-NEU; P-CARD; NEO; GS-14F; PED-OPD; P-PULM-W1; P-GI; A-ICU; P-PULM-W2; PICU; P-HEME; URO-24F
C	Ascensia CONTOUR TS (Ascensia Diabetes Care, Switzerland)	(n=2), TCM; P-NRI
D	AU5800 (Beckman Coulter Diagnostics, Brea, CA, USA)	(n=1), CL

Abbreviations: RM = Reproductive Medicine; ORTH = Orthopedics; OB-xF = Obstetrics (xth floor); OB-OPD = Obstetrics Outpatient Department; IM-25F = Internal Medicine (25th floor); IMed = Integrative Medicine; GYN-xF = Gynecology (xth floor); TBS = Thyroid & Breast Surgery; ENT = Otolaryngology; ED = Emergency Department; GS-xF = General Surgery (xth floor); ANES/OR = Anesthesiology & Operating Room; P-NEU = Pediatric Neurology; P-CARD = Pediatric Cardiology; NEO = Neonatology; PED-OPD = Pediatrics Outpatient Department; P-PULM-W1 = Pediatric Pulmonology, Ward 1; P-PULM-W2 = Pediatric Pulmonology, Ward 2; P-GI = Pediatric Gastroenterology; A-ICU = Adult Intensive Care Unit; PICU = Pediatric Intensive Care Unit; P-HEME = Pediatric Hematology; URO-24F = Urology (24th floor); TCM = Traditional Chinese Medicine; P-NRI = Pediatric Nephrology–Rheumatology–Immunology; CL = Central Laboratory (Core Lab).

Table S2. Brand-level accuracy and agreement with laboratory reference (mg/dL).

Brand	Pairs (n)	ISO 15197 %, [95% CI]	MARD, % [95% CI]	RMSE, mg/dL [95% CI]	BA mean bias, mg/dL (95% LoA)	LoA width, mg/dL [95% CI]
Ascensia	10	100.0 [72.2, 100.0]	5.0 [4.9, 5.2]	10.3 [9.4, 11.2]	-7.45 (-22.28, 7.38)	29.7 [27.2, 31.5]
LifeScan	105	100.0 [96.5, 100.0]	3.7 [3.1, 4.4]	7.6 [6.4, 8.6]	-0.68 (-15.61, 14.24)	29.9 [25.0, 34.2]
Roche	60	100.0 [94.0, 100.0]	3.8 [3.3, 4.4]	6.3 [5.6, 6.8]	-1.72 (-13.62, 10.18)	23.8 [20.9, 25.9]

Notes. 95% CIs by percentile bootstrap (R = 2000). Bland–Altman as mean bias and 95% LoA; LoA width CI conservatively derived from bounds. Ascensia n = 10: exploratory estimates due to limited precision.

Table S3. Per-level analytical performance of POCT glucose versus the laboratory reference (35 instruments; one per department; three brands).

Reference level (mg/dL)	n	ISO 15197 within limits (%)	ISO 95% CI (%)	Mean_Bias (mg/dL)	Bias 95% CI (mg/dL)	RMSE (mg/dL)	RMSE 95% CI (mg/dL)	MARD (%)	MARD 95% CI (%)
291	35	100	90.1, 100.0	−11.34	−13.45, −8.92	13.34	11.45, 15.13	4.18	3.58, 4.80
227	35	100	90.1, 100.0	0.00	−2.26, 2.16	6.79	5.11, 8.33	2.29	1.68, 2.98
123	35	100	90.1, 100.0	1.08	−0.62, 2.62	5.09	3.38, 6.66	2.81	1.83, 3.80
82	35	100	90.1, 100.0	2.11	1.18, 2.98	3.40	2.77, 3.99	3.36	2.53, 4.17
41	35	100	90.1, 100.0	1.03	-0.05, 1.90	3.17	2.31, 4.09	5.37	5.03, 7.94

Metrics were computed from instrument-level means of triplicates at each reference level (n = 35 per level). ISO 15197 within limits (%) denotes the proportion within ±15 mg/dL for Ref < 100 mg/dL or within ±15% for Ref ≥ 100 mg/dL. Let Δ = POCT − Ref (mg/dL). Bias (mg/dL) is mean(Δ); RMSE (mg/dL) is $\sqrt{\text{mean}(\Delta^2)}$; MARD (%) is mean($|\Delta|$/Ref × 100). 95% confidence intervals (CIs) were calculated as follows: ISO proportions by the Wilson method; Bias, RMSE, and MARD by percentile bootstrap (B = 2000). Units are mg/dL (mmol/L ≈ mg/dL ÷ 18).

Table S4. Department-level *r*-based performance rankings at five reference levels

Table S4a. Department-level performance at 41mg/dl glucose reference level

Department	Rank	*r*	Risk	*r**TEa	*r**1/2TEa
Emergency Department	1	0.328	High	0.748	0.874
Neonatology	2	0.568	High	0.748	0.874
Traditional Chinese Medicine	3	0.688	High	0.748	0.874
Gynecology (17th floor)	4	0.712	High	0.748	0.874
Integrative Medicine	5	0.712	High	0.748	0.874
Obstetrics (9th floor)	6	0.712	High	0.748	0.874
Pediatric Nephrology-Rheumatology-Immunology	7	0.808	Medium	0.748	0.874
Obstetrics Outpatient Department	8	0.832	Medium	0.748	0.874
Pediatric Intensive Care Unit	9	0.832	Medium	0.748	0.874
Adult Intensive Care Unit	10	0.832	Medium	0.748	0.874
Anesthesiology & Operating Room	11	0.832	Medium	0.748	0.874
General Surgery (14th floor)	12	0.832	Medium	0.748	0.874
General Surgery (21th floor)	13	0.832	Medium	0.748	0.874
Gynecology (18th floor)	14	0.8312	Medium	0.748	0.874
Obstetrics (10th floor)	15	0.832	Medium	0.748	0.874
Obstetrics (7th floor)	16	0.832	Medium	0.748	0.874
Obstetrics (8th floor)	17	0.832	Medium	0.748	0.874
Pediatric Cardiology	18	0.832	Medium	0.748	0.874
Pediatric Gastroenterology	19	0.832	Medium	0.748	0.874
Pediatric Hematology	20	0.832	Medium	0.748	0.874
Pediatric Neurology	21	0.832	Medium	0.748	0.874
Pediatric Pulmonology, Ward 2	22	0.832	Medium	0.748	0.874
Pediatrics Outpatient Department	23	0.832	Medium	0.748	0.874
Pediatric Pulmonology, Ward 1	24	0.832	Medium	0.748	0.874
Urology (24th floor)	25	0.832	Medium	0.748	0.874
Internal Medicine (25th floor)	26	0.928	Medium	0.748	0.874
Otolaryngology	27	0.928	Low	0.748	0.874
Thyroid & Breast Surgery	28	0.928	Low	0.748	0.874
Obstetrics (20th floor)	29	0.952	Low	0.748	0.874
Obstetrics (21th floor)	30	0.952	Low	0.748	0.874
Obstetrics (22th floor)	31	0.952	Low	0.748	0.874
Reproductive Medicine	32	0.952	Low	0.748	0.874
Gynecology (16th floor)	33	0.952	Low	0.748	0.874
Gynecology (19th floor)	34	0.952	Low	0.748	0.874
Orthopedics	35	0.952	Low	0.748	0.874

Table S4b. Department-level performance at 82mg/dl glucose reference level

Department	Rank	*r*	Risk	*r**TEa	*r**1/2TEa
Emergency Department	1	0.544	High	0.617	0.809
General Surgery (14th floor)	2	0.616	High	0.617	0.809
Pediatric Gastroenterology	3	0.616	High	0.617	0.809
Adult Intensive Care Unit	4	0.616	High	0.617	0.809
Gynecology (19th floor)	5	0.616	High	0.617	0.809
Pediatrics Outpatient Department	6	0.616	High	0.617	0.809
General Surgery (21th floor)	7	0.616	High	0.617	0.809
Integrative Medicine	8	0.736	Medium	0.617	0.809
Obstetrics (22th floor)	9	0.736	Medium	0.617	0.809
Obstetrics (8th floor)	10	0.736	Medium	0.617	0.809
Pediatric Hematology	11	0.736	Medium	0.617	0.809
Pediatric Neurology	12	0.736	Medium	0.617	0.809
Pediatric Pulmonology, Ward 1	13	0.736	Medium	0.617	0.809
Pediatric Nephrology-Rheumatology-Immunology	14	0.784	Medium	0.617	0.809
Gynecology (17th floor)	15	0.856	Low	0.617	0.809
Obstetrics (10th floor)	16	0.856	Low	0.617	0.809
Internal Medicine (25th floor)	17	0.856	Low	0.617	0.809
Pediatric Intensive Care Unit	18	0.856	Low	0.617	0.809
Gynecology (16th floor)	19	0.856	Low	0.617	0.809
Gynecology (18th floor)	20	0.856	Low	0.617	0.809
Obstetrics (20th floor)	21	0.856	Low	0.617	0.809
Obstetrics (21th floor)	22	0.856	Low	0.617	0.809
Pediatric Pulmonology, Ward 2	23	0.856	Low	0.617	0.809
Reproductive Medicine	24	0.856	Low	0.617	0.809
Obstetrics Outpatient Department	25	0.856	Low	0.617	0.809
Neonatology	26	0.904	Low	0.617	0.809
Anesthesiology & Operating Room	27	0.976	Low	0.617	0.809
Thyroid & Breast Surgery	28	0.976	Low	0.617	0.809
Obstetrics (7th floor)	29	0.976	Low	0.617	0.809
Obstetrics (9th floor)	30	0.976	Low	0.617	0.809
Pediatric Cardiology	31	0.976	Low	0.617	0.809
Traditional Chinese Medicine	32	0.976	Low	0.617	0.809
Urology (24th floor)	33	0.976	Low	0.617	0.809
Orthopedics	34	0.976	Low	0.617	0.809
Otolaryngology	35	0.976	Low	0.617	0.809

Table S4c. Department-level performance at 123 mg/dl glucose reference level

Department	Rank	*r*	Risk	*r**TEa	*r**1/2TEa
Neonatology	1	0.2	High	0.533	0.767
Gynecology (18th floor)	2	0.434	High	0.533	0.767
Integrative Medicine	3	0.434	High	0.533	0.767
Internal Medicine (25th floor)	4	0.434	High	0.533	0.767
Obstetrics (10th floor)	5	0.532	High	0.533	0.767
Obstetrics (9th floor)	6	0.532	High	0.533	0.767
Obstetrics (21th floor)	7	0.532	High	0.533	0.767
Urology (24th floor)	8	0.59	Medium	0.533	0.767
Adult Intensive Care Unit	9	0.785	Low	0.533	0.767
Obstetrics (8th floor)	10	0.824	Low	0.533	0.767
Pediatric Gastroenterology	11	0.883	Low	0.533	0.767
Pediatric Pulmonology, Ward 2	12	0.883	Low	0.533	0.767
Obstetrics Outpatient Department	13	0.883	Low	0.533	0.767
Pediatric Hematology	14	0.883	Low	0.533	0.767
Pediatric Intensive Care Unit	15	0.883	Low	0.533	0.767
Pediatric Nephrology-Rheumatology-Immunology	16	0.883	Low	0.533	0.767
Orthopedics	17	0.922	Low	0.533	0.767
Anesthesiology & Operating Room	18	0.922	Low	0.533	0.767
General Surgery (21th floor)	19	0.922	Low	0.533	0.767
Gynecology (16th floor)	20	0.922	Low	0.533	0.767
Gynecology (17th floor)	21	0.922	Low	0.533	0.767
Gynecology (19th floor)	22	0.922	Low	0.533	0.767
Obstetrics (20th floor)	23	0.922	Low	0.533	0.767
Obstetrics (22th floor)	24	0.922	Low	0.533	0.767
Pediatric Cardiology	25	0.922	Low	0.533	0.767
Thyroid & Breast Surgery	26	0.922	Low	0.533	0.767
Traditional Chinese Medicine	27	0.922	Low	0.533	0.767
General Surgery (14th floor)	28	0.922	Low	0.533	0.767
Pediatric Pulmonology, Ward 1	29	0.922	Low	0.533	0.767
Pediatric Neurology	30	0.98	Low	0.533	0.767
Emergency Department	31	0.98	Low	0.533	0.767
Obstetrics (7th floor)	32	0.98	Low	0.533	0.767
Otolaryngology	33	0.98	Low	0.533	0.767
Pediatrics Outpatient Department	34	0.98	Low	0.533	0.767
Reproductive Medicine	35	0.981	Low	0.533	0.767

Table S4d. Department-level performance at 227mg/dl glucose reference level

Department	Rank	*r*	Risk	*r**TEa	*r**1/2TEa
Otolaryngology	1	0.567	Medium	0.533	0.767
Pediatric Nephrology-Rheumatology-Immunology	2	0.567	Medium	0.533	0.767
Traditional Chinese Medicine	3	0.567	Medium	0.533	0.767
Emergency Department	4	0.588	Medium	0.533	0.767
Orthopedics	5	0.641	Medium	0.533	0.767
Thyroid & Breast Surgery	6	0.672	Medium	0.533	0.767
General Surgery (21th floor)	7	0.746	Medium	0.533	0.767
Obstetrics (9th floor)	8	0.746	Medium	0.533	0.767
Gynecology (16th floor)	9	0.799	Low	0.533	0.767
Pediatric Gastroenterology	10	0.831	Low	0.533	0.767
Pediatric Pulmonology, Ward 1	11	0.852	Low	0.533	0.767
General Surgery (14th floor)	12	0.852	Low	0.533	0.767
Obstetrics (7th floor)	13	0.852	Low	0.533	0.767
Obstetrics Outpatient Department	14	0.852	Low	0.533	0.767
Reproductive Medicine	15	0.852	Low	0.533	0.767
Integrative Medicine	16	0.884	Low	0.533	0.767
Gynecology (18th floor)	17	0.884	Low	0.533	0.767
Pediatrics Outpatient Department	18	0.884	Low	0.533	0.767
Adult Intensive Care Unit	19	0.884	Low	0.533	0.767
Neonatology	20	0.905	Low	0.533	0.767
Obstetrics (20th floor)	21	0.905	Low	0.533	0.767
Pediatric Neurology	22	0.905	Low	0.533	0.767
Pediatric Cardiology	23	0.936	Low	0.533	0.767
Gynecology (17th floor)	24	0.937	Low	0.533	0.767
Gynecology (19th floor)	25	0.937	Low	0.533	0.767
Internal Medicine (25th floor)	26	0.937	Low	0.533	0.767
Urology (24th floor)	27	0.937	Low	0.533	0.767
Obstetrics (21th floor)	28	0.937	Low	0.533	0.767
Obstetrics (10th floor)	29	0.958	Low	0.533	0.767
Obstetrics (22th floor)	30	0.958	Low	0.533	0.767
Obstetrics (8th floor)	31	0.958	Low	0.533	0.767
Pediatric Intensive Care Unit	32	0.958	Low	0.533	0.767
Anesthesiology & Operating Room	33	0.989	Low	0.533	0.767
Pediatric Pulmonology, Ward 2	34	0.989	Low	0.533	0.767
Pediatric Hematology	35	0.990	Low	0.533	0.767

Table S4e. Per-department performance at glucose reference level 291 mg/dL

Department	Rank	*r*	Risk	*r**TEa	*r**1/2TEa
Anesthesiology & Operating Room	1	0.468	High	0.533	0.767
Obstetrics (10th floor)	2	0.509	High	0.533	0.767
Reproductive Medicine	3	0.509	High	0.533	0.767
General Surgery (21th floor)	4	0.509	High	0.533	0.767
Pediatric Nephrology-Rheumatology-Immunology	5	0.551	Medium	0.533	0.767
Gynecology (18th floor)	6	0.592	Medium	0.533	0.767
Orthopedics	7	0.592	Medium	0.533	0.767
Obstetrics (9th floor)	8	0.633	Medium	0.533	0.767
Pediatric Pulmonology, Ward 2	9	0.633	Medium	0.533	0.767
Emergency Department	10	0.666	Medium	0.533	0.767
Obstetrics (21th floor)	11	0.674	Medium	0.533	0.767
Traditional Chinese Medicine	12	0.674	Medium	0.533	0.767
Urology (24th floor)	13	0.674	Medium	0.533	0.767
Adult Intensive Care Unit	14	0.715	Medium	0.533	0.767
Obstetrics Outpatient Department	15	0.715	Medium	0.533	0.767
Otolaryngology	16	0.715	Medium	0.533	0.767
Pediatric Cardiology	17	0.715	Medium	0.533	0.767
Pediatric Gastroenterology	18	0.715	Medium	0.533	0.767
Pediatric Intensive Care Unit	19	0.715	Medium	0.533	0.767
Pediatric Hematology	20	0.716	Medium	0.533	0.767
Internal Medicine (25th floor)	21	0.757	Medium	0.533	0.767
Obstetrics (22th floor)	22	0.757	Medium	0.533	0.767
Pediatric Neurology	23	0.757	Medium	0.533	0.767
Pediatric Pulmonology, Ward 1	24	0.757	Medium	0.533	0.767
Pediatrics Outpatient Department	25	0.798	Low	0.533	0.767
Thyroid & Breast Surgery	26	0.798	Low	0.533	0.767
Integrative Medicine	27	0.839	Low	0.533	0.767
Neonatology	28	0.839	Low	0.533	0.767
Obstetrics (7th floor)	29	0.839	Low	0.533	0.767
Gynecology (17th floor)	30	0.880	Low	0.533	0.767
Gynecology (19th floor)	31	0.880	Low	0.533	0.767
Obstetrics (20th floor)	32	0.880	Low	0.533	0.767
Obstetrics (8th floor)	33	0.880	Low	0.533	0.767
Gynecology (16th floor)	34	0.922	Low	0.533	0.767
General Surgery (14th floor)	35	0.963	Low	0.533	0.767
